# *In Vitro* Neutral Detergent Cellulase Method and Chemical Composition to Predict *In Vivo* Fermentable Organic Matter of Roughages

**DOI:** 10.3390/ani11061594

**Published:** 2021-05-28

**Authors:** Yue Liu, Rui Li, Hao Wu, Qingxiang Meng, Muhammad Zahoor Khan, Zhenming Zhou

**Affiliations:** State Key Laboratory of Animal Nutrition, College of Animal Science and Technology, China Agricultural University, Beijing 100193, China; yueliu@cau.edu.cn (Y.L.); lirui3822@yeah.net (R.L.); wu2213@cau.edu.cn (H.W.); qxmeng@cau.edu.cn (Q.M.); zahoorcau@cau.edu.cn (M.Z.K.)

**Keywords:** *in situ* nylon bag technique, *in vitro* neutral detergent cellulase plus amylase method, fermentable organic matter

## Abstract

**Simple Summary:**

Various methods such as *in situ*, gas production and enzymatic methods are exercised to estimate the *in vivo* fermentable organic matter (FOM). However, each of these methods has its limitations. The *in vivo* method with fistulated animals for FOM determination is expensive, laborious and negatively affects animal welfare. Similarly, the *in situ* method also requires rumen fluid and is costly. However, enzymatic methods eliminate the need for fistulated animals and are comparatively simple, cheaper, faster, have greater repeatability, and also ensure the standardization of the process. Additionally, *in situ* technique can be disregarded as a standard method to test the accuracy of other techniques in cases where *in vivo* testing is not feasible. Therefore, in the current study, we compared the *in situ* nylon bag technique with the *in vitro* neutral detergent cellulase method and chemical composition to estimate *in vivo* FOM of roughages.

**Abstract:**

*In Vivo* fermentable organic matter (FOM) reflects the energy production and the potential of rumen’s microbial protein synthesis. However, the *in vivo* method with fistulated animals for FOM measurement compromises animal welfare and is laborious as well as expensive. Although the alternative *in situ* nylon bag technique has been widely used, it is also costly and requires rumen liquor. Therefore, the present study was performed to compare the *in situ* nylon bag technique with the *in vitro* neutral detergent cellulase (NDC) method or chemical composition to estimate *in vivo* FOM of roughages. For this purpose, we selected 12 roughages, including six each from forages and crop residues. Our results have shown the strong correlation equations between FOM*_in situ_* and FOM_NDC_ of forages (n = 6; R^2^ = 0.79), crop residues (n = 6; R^2^ = 0.80), and roughages (n = 12; R^2^ = 0.84), respectively. Moreover, there were also strong correlations between the chemical composition of roughages and FOM*_in situ_* (n = 12; R^2^ = 0.84–0.93) or FOM_NDC_ (n = 12; R^2^ = 0.79–0.89). In conclusion, the *in vitro* NDC method and chemical composition were alternatives to *in situ* nylon bag technique for predicting *in vivo* FOM of roughages in the current experiment.

## 1. Introduction

*In Vivo* fermentable organic matter (FOM) of roughages reflects the rumen’s energy production and the potential of microbial protein synthesis [1]. The determination of *in vivo* FOM of forages with fistulated animals is costly, time-consuming, and negatively affects animal welfare [2]. On the other hand, in the French small intestine digestible protein system, FOM is calculated from organic matter total tract digestibility (OMD) [3,4].

Previous studies have shown that the methods of *in situ* nylon bag, gas production, pepsin–cellulase (PC), Tilley and Terry, and neutral detergent cellulase (NDC) are well correlated with *in vivo* OMD [5,6,7,8,9]. Although the *in situ* nylon bag technique has been widely used, this method requires fistulated animals and is also expensive [5]. Other existing *in vitro* methods, such as Tilley and Terry [5] and gas production [7], also need rumen liquor. Compared to rumen liquor-based methods, enzymatic methods eliminate the need for fistulated animals, are simpler, cheaper, faster, have greater repeatability, and ensure the standardization of the process [1,9]. Moreover, the PC method requires regular evaluation to ensure accurate results, while the NDC method has not been updated [6] and is faster than the pretreatment of samples with pepsin [10]. Therefore, it seems that NDC method may be an attractive alternative for predicting *in vivo* FOM. Moreover, it was shown that *in vitro* digestibility determined by rumen fluid or enzymes was superior to chemical characteristics [5,11]; however, the chemical composition method is considered the simpler, faster, and cheaper method [12]. Thus, the chemical composition method has also attempted to predict *in vivo* FOM in the current study. Kitessa et al. [3] reported that the *in situ* nylon bag technique could be disregarded as a standard method to test the accuracy of other techniques in cases where *in vivo* testing is not feasible. Additionally, the *in situ* nylon bag technique with the Bang-Bang (BB) apparatus was inexpensive, portable, and easy to operate to measure the rumen degradation characteristics of feedstuffs than traditional steel chain or flexible plastic tubes for binding the bags used in the *in situ* nylon bag technique [13]. Therefore, in the current study, we considered the *in situ* nylon bag technique with the BB apparatus as the reference method.

To our knowledge, no study has performed an *in vitro* NDC method to predict *in vivo* FOM, and the correlation of chemical composition to predict *in vivo* FOM is also controversial and unclear. This study aimed to determine the possibility of predicting *in vivo* FOM of roughages using the *in vitro* NDC method or chemical composition. Thus, we hypothesized that the *in vitro* NDC method and chemical composition are suitable methods for an alternative *in vivo* FOM of roughages.

## 2. Materials and Methods

### 2.1. Sample Collection

Twelve roughage samples, including six each from forages and crop residues, were collected from five main beef cattle breeding areas in China. Specifically, the samples were langsdorff small reed (Hulunbuir City, Inner Mongolia), alfalfa, avena nuda straw, corn straw (Zuoyun County, Shanxi Province), mixed forage, oat straw, hulless barley straw, rapeseed straw (Hezuo City, Gansu Province), lolium perenne (Xiahe County, Gansu Province), *poa annua* (Nagqu Prefecture, Tibet Autonomous Region), *alpine kobresia* (Maizhokunggar County, Tibet Autonomous Region), and barley straw (Haiyan County, Qinghai Province). Mixed forage was collected from the natural meadow. Three samples were selected from each forage and crop residue for further analysis.

### 2.2. Chemical Analysis

The samples were oven-dried at 55 °C for 48 h and ground to a 2-mm mesh-screen size using a feed mill (DF-20, Wenling Linda Machinery Co. Ltd., Wenling, Zhejiang, China). Dry matter (DM; method 930.15) and ash (method 942.05) were analyzed by AOAC methods (2000) [14]. Nitrogen was determined using a protein analyzer (Rapid N III, Elementar Inc., Germany), and CP was calculated as the percentage of nitrogen × 6.25. Further, crude fiber (CF), neutral detergent fiber (NDF), and acid detergent fiber (ADF) were determined using a fiber analyzer (ANKOM A220, ANKOM Technology Corp., Macedon, NY, USA). Ether extract (EE) was extracted using an automatic extractor (ANKOM XT101, ANKOM Technology Corp., Macedon, NY, USA). Nitrogen-free extract (NFE) was calculated using the formula: w(NFE) = w(DM) − w(CP) – w(EE) – w(CF) – w(Ash). The chemical compositions and digestibility of each sample were calculated in triplicate.

### 2.3. In Situ Nylon Bag Technique

Three Angus steers fitted with a permanent rumen cannula (450 ± 15 kg) were utilized to determine the effective rumen degradability of the organic matter of roughages with the nylon bag technique within the BB apparatus [13]. Animals were fed a total mixed ration at 8:00 h and 16:00 h according to NRC 2000 to meet the ME requirement for 1.3 × maintenance [15], and ad libitum access to water and a mineral block was provided. This animal experiment was approved by the China Agricultural University Animal Care and Use Committee (AW28059102-1, Beijing, China). The sample was weighed (4.00 ± 0.01 g) in nylon bags (80 × 140 mm) with a pore size of 37 µm, and then suspended in the rumen before morning feeding. Bags were removed after 6, 24, 48, 72, 96, 120, and 144 h of incubation. After removal, the bags were immediately immersed in cold water to stop fermentation, washed six times (1 min/rinse) in a washing machine until the water was clean, then dried at 65 °C for 48 h. Rumen degradation kinetics was determined using the following equations.

Rumen dynamic degradation rate: y = a + b (1 − *e^−ct^)* (1)

Effective degradability: ED = a + (b × c) / (c + k) (2)

Here, y is the rumen degradation rate at time t, a, b, c, t, and k stand for the rapidly degraded fraction (%), the slowly degraded fraction (%), the degradation rate constant at which b is degraded (%/h), incubation time, and rumen passage rate at 0.0253%/h, respectively. The effective degradability (ED) of organic matter was regarded as the rumen fermentable organic matter (FOM*_in situ_*) [16].

### 2.4. In Vitro Neutral Detergent-Cellulase Plus Amylase Method

The *in vitro* organic matter digestibility was determined by NDC plus amylase method using small nylon bags (37 µm, 25 × 60 mm). Samples (0.50 ± 0.001 g) were transferred to small nylon bags, sealed (FR-300B, Blueberry, Shanghai, China), boiled in neutral detergent solution with heat-stable α-amylase for 75 min (ANKOM A220, ANKOM Technology Corp., Macedon, NY, USA), then oven-dried at 65 °C for 48 h. For the cellulase buffer solution preparation, we incubated 20 g cellulase [17] in a 1 L acetic acid buffer (pH 4.8) for 1 h at 40 °C. Two small nylon bags were randomly placed in 100-mL culture tubes and pre-warmed overnight at 39 °C. The next morning, cellulase buffer solution (80 mL) was added into each preheated 100-mL culture tube using an automatic pump and then incubated for 24 h in a 40 ± 2 °C water at 40–60 rpm (DSHZ-300A, Jiangsu, China). Following incubation, the bags were immersed in cold water to stop fermentation, washed as *in situ* nylon bag technique, and oven-dried at 65 °C for 48 h.

### 2.5. Statistical Analysis

We analyzed the chemical composition and *in situ* organic matter degradation characteristics of samples using a two-tailed Student’s t-test [18]. Then we compared the FOM*_in situ_* and FOM_NDC_ of samples using the general linear model (GLM) procedure of SAS version 9.0 (SAS Institute Inc., Cary, NC, USA): y_ij_ = μ + a_i_ + e_ij_, where y_ij_ is *j*th observation value at the ith level of factor a, μ is the population mean, a_i_ is the treatment effect at level i of factor a, and e_ij_ is the individual random residual error. The data of the prediction equations were analyzed using the linear regression procedure (REG) of SAS version 9.0 (SAS Institute Inc., Cary, NC, USA): y = b_0_ + b_1_x_1_ + b_2_x_2_ + ⋯ + b_m_x_m_, where y is the dependent variable and x_1_, x_2_, x_3_, ⋯ x_m_represent m independent variables. Statistical significance was set at *p* < 0.05.

## 3. Results

### 3.1. Chemical Compositions of Roughages

The data regarding chemical analysis for contents (%) of DM, OM, EE, CP, CF, NDF, ADF, Ash, and NFE are presented in Table 1. Compared with crop residues, EE, CP and NFE of forages were higher (*p* < 0.01), but CF, NDF, and ADF were lower (*p* < 0.01).

### 3.2. In Situ Organic Matter Degradation

*In Situ* organic matter degradation characteristics of roughages are shown in Table 2. Compared with crop residues, a (*p* < 0.01), b (*p* < 0.01), c (*p* = 0.01), and FOM*_in situ_* (*p* < 0.01) were higher in the forages.

### 3.3. In Situ Organic Matter Disappearance Rate

**Figure 1:**
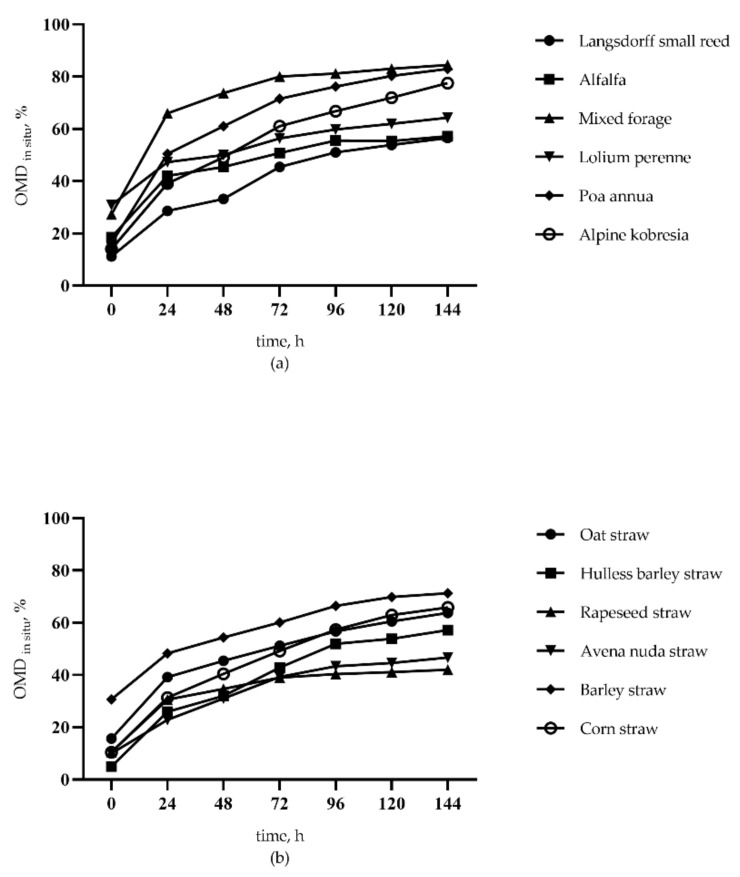
Incubated time (x) and in situ organic matter disappearance rate (y) of forages (**a**) and crop residues (**b**), respectively.

*In Situ* organic matter disappearance rate (OMD*_in situ_*) of forages (a) and crop residues (b) are demonstrated in Figure 1. In brief, our data show that at 144 h, OMD*_in situ_* of forages from high to low were mixed forage (84.82), *poa annua* (82.92%), *alpine kobresia* (77.55%), lolium perenne (64.24%), alfalfa (57.21%), and langsdorff small reed (56.23%). Consequently, OMD*_in situ_* of crop residues were 71.27% in barley straw, 65.88% in corn straw, 63.77% in oat straw, 57.91% in hulless barley straw, 46.72% in avena nuda straw, and 42.08% in rapeseed straw.

### 3.4. Prediction Equations of FOM_in situ_ Based on Chemical Composition

The regression equations between FOM*_in situ_* and chemical composition (EE, CP, CF, NDF, ADF, Ash, and NFE) of roughages (n = 12) are presented in Table 3. From equations 1 to 5 achieved higher reliability (*p* < 0.01), which contain CF (R^2^ = 0.84), ADF (R^2^ = 0.87), EE and CF (R^2^ = 0.92), EE, CP and ADF (R^2^ = 0.92), EE, CP, and CF (R^2^ = 0.93).

### 3.5. Comparison of FOM_NDC_ and FOM_in situ_

The FOM_NDC_ and FOM*_in situ_* were presented in Table 4. For forages and crop residues, we only obtained a difference in alfalfa (*p* = 0.02).

### 3.6. Correlation Analysis between FOM_NDC_ and FOM_in situ_ of Forages, Crop Residues, and Roughages

**Figure 2:**
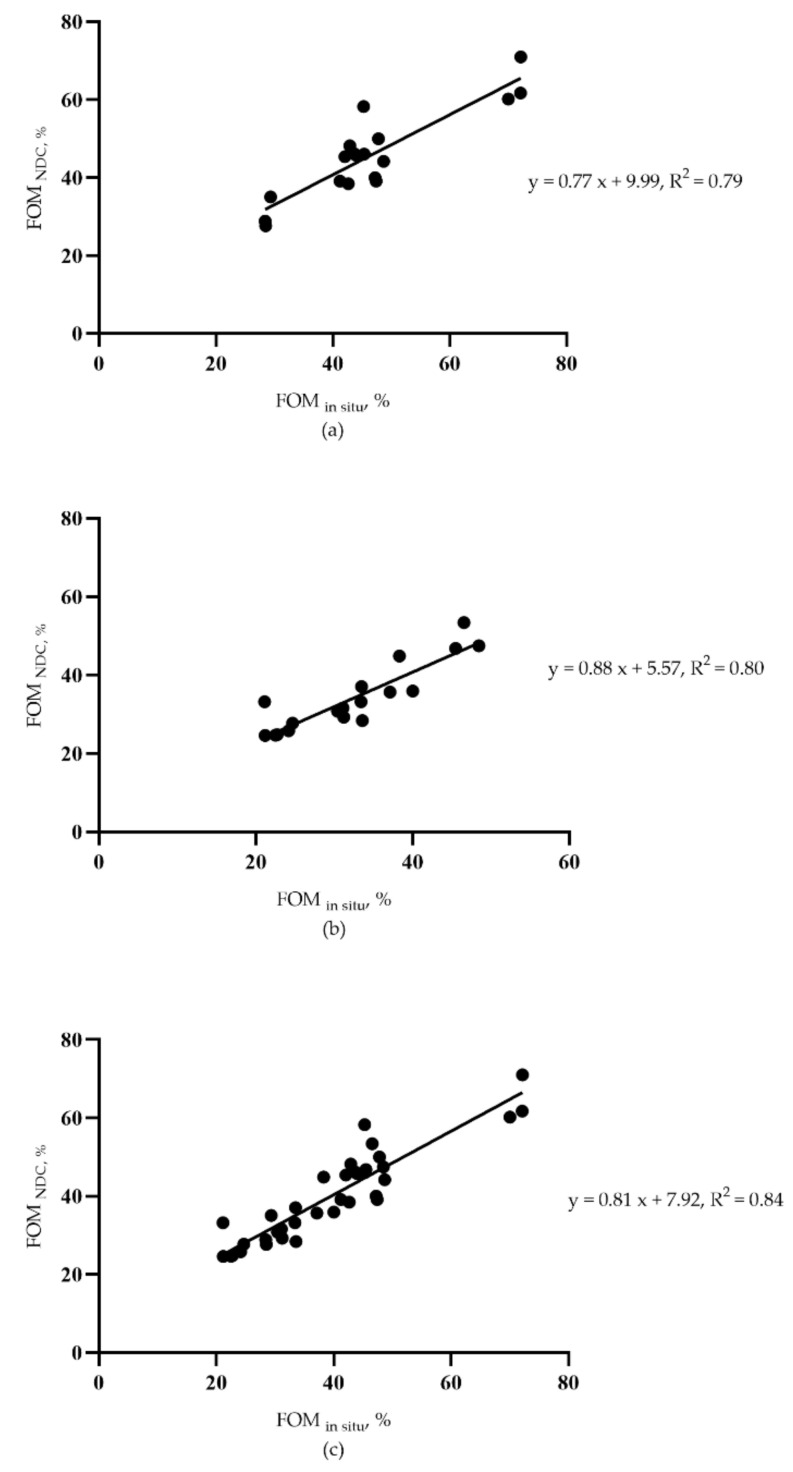
Linear equations of fermentable organic matter between determined by the *in vitro* neutral detergent–cellulase plus amylase method (x) and the *in situ* nylon bag technique (y) among forages (**a**), crop residues (**b**) and roughages (**c**), respectively.

The correlation analysis between FOM_NDC_ and FOM*_in situ_* of forages (a), crop residues (b), and roughages (c) is presented in Figure 2. Our results show the strong correlation equations between FOM*_in situ_* and FOM_NDC_ of forages (n = 6; R^2^ = 0.79), crop residues (n = 6; R^2^ = 0.80), and roughages (n = 12; R^2^ = 0.84), respectively.

### 3.7. Prediction Equations of FOM_NDC_ Based on Chemical Composition

The regression equations generated between FOM_NDC_ and chemical composition (EE, CP, CF, NDF, ADF, Ash, and NFE) of roughages are presented in Table 5. Based on our findings, it was shown that the R^2^ increased in equations 1 through 8 (*p* < 0.01), which contains a chemical composition of CF (R^2^ = 0.79), EE and NDF (R^2^ = 0.83), ADF (R^2^ = 0.84), EE, CP and NDF (R^2^ = 0.84), EE and ADF (R^2^ = 0.84), EE, CP and ADF (R^2^ = 0.88), EE, CP, CF (R^2^ = 89), EE and CF (R^2^ = 0.89), respectively.

## 4. Discussion

*In Vivo* FOM depends on rumen dynamic processes, while *in vivo* OMD depends on the digestion of OM in the total digestive tract, and a reduction in the rumen can be compensated with the enhancement of the fermentation of the hindgut. Therefore, *in* vivo FOM’s determination is more difficult than that of *in vivo* OMD; the measurement of *in vivo* FOM is also less precise than *in vivo* OMD. Consistently, the differences between alternative methods will probably be more pronounced when correlated with *in vivo* FOM than with *in vivo* OMD [1].

It was shown in a previous report that the *in vitro* method is the most inaccurate in predicting the digestibility of some low-quality feeds, such as hay and grain by-products [3]. The low-quality feed has higher contents of insoluble material, which up to some extent, determines the digestibility of a feed. When the roughage is treated with neutral detergent solution, the soluble carbohydrates, pectin, proteins, and other soluble components dissolve, leaving insoluble cell walls, which can be degraded by cellulase [10]. Therefore, in our study, we selected the NDC method. Consequently, to combine the characteristics of beef roughage, we used low-quality roughages as experimental material, which is in line with previous findings [19].

Although studies have suggested that the feedstuff’s chemical composition cannot be used to predict OMD satisfactorily [3,20,21]. On the contrary, a good relationship between chemical composition and FOM*_in situ_* or FOM_NDC_ of roughages was observed in the current study. These findings suggest that it is possible to predict *in vivo* FOM by the chemical compositions of a sample. Moreover, research has shown that chemical composition is related to DMI, DMI is negatively related to NDF, and NDF associated with gastrointestinal filling [22], and a strong relationship between NDF and ADF is well established, which can explain that equations based on ADF and NDF are accurate enough to predict FOM*_in situ_* or FM_NDC_ of roughages. Additionally, the prediction of EE, CP, and CF in the same equation was more accurate than that of CF or ADF alone, suggesting that using chemical composition to predict *in vivo* FOM may be more correlated with fiber components. In the future, with increasing the number of samples, more accurate prediction equations are expected to be obtained.

The findings of our study showed a strong correlation between FOM_NDC_ and FOM*_in situ_* of roughages (R^2^ = 0.84). Consistently, Mary et al. [10] had documented the correlation between NDC and *in vivo* values and seemed to be consistent with those obtained by Tilley and Terry techniques. Moreover, that relationship was increased with the number of samples. Givens et al. [23] also reported a higher R^2^ value between *in vivo* OMD and *in vitro* OMD in the spring forage than in the autumn forage. Future samples collected only once to construct the equation will not be adequate; the collection of more samples at different times of the year is suggested.

It may be noted that enzyme-based FOM measurements did not take into account possible interactions between microbial species present in the rumen. Although the nylon bag technique feed samples are digested in the actual rumen environment, many of the procedures involved in this method have not been standardized, and the range of variability within laboratories may be greater than all other *in vitro* methods [3]. Therefore, different methods have their advantages and disadvantages, but as long as the correlation between methods is strong, they might be replaced by each other.

## 5. Conclusions

Altogether, it was concluded that there is a strong relationship between FOM values obtained with the in situ nylon bag technique and the in vitro NDC method, which FOM*_in situ_* = 0.77 FOM_NDC_ + 9.90 (forages, R^2^ = 0.79), FOM*_in situ_* = 0.88 FOM_NDC_ + 5.57 (crop residues, R^2^ = 0.80), FOM*_in situ_* = 0.81 FOM_NDC_ + 7.92 (roughages, R^2^ = 0.84). Moreover, the FOM predicted by chemical composition correlates very well with the in situ nylon bag technique (R^2^ = 0.84–0.93) and in vitro NDC method (R^2^ = 0.79–0.89), respectively. Therefore, the NDC method and chemical composition seem adequate to develop equations to predict the in vivo FOM. However, more research is warranted to develop local equations considering specific pasture types, environmental conditions such as harvest season, and phenological stages, and to corroborate such predictions with in vivo data where conditions permit.

## Figures and Tables

**Table 1:** Chemical compositions of roughages (DM basis, expressed as %).

Item	Roughages	DM	OM	EE	CP	CF	NDF	ADF	Ash	NFE
Forages	Langsdorff small reed	93.28	94.67	0.84	5.94	38.25	74.88	42.66	5.33	42.91
	Alfalfa	92.40	94.10	0.65	18.40	29.03	42.60	34.43	5.90	42.17
	Mixed forage	92.62	91.15	2.02	14.85	22.19	45.43	25.15	8.85	44.71
	Lolium perenne	91.01	95.36	0.68	6.85	32.16	61.84	37.91	4.64	44.69
	*Poa annua L.*	93.29	93.03	1.47	14.59	30.36	69.08	33.85	6.97	39.90
	*Alpine kobresia*	92.80	95.73	1.27	12.80	34.80	73.82	36.17	4.27	39.67
Mean		92.57	94.00	1.16 ^a^	12.24 ^a^	31.13 ^b^	61.28 ^b^	35.03 ^b^	6.00	42.68 ^a^
Crop residues	Oat straw	93.08	90.56	0.81	12.56	35.48	68.84	40.75	9.44	34.80
	Hulless barley straw	92.52	92.53	0.63	4.13	41.22	82.64	51.47	7.47	39.07
	Rapeseed straw	91.27	91.96	1.46	9.96	48.26	70.65	52.20	8.04	23.55
	Avena nuda straw	93.14	94.01	0.67	2.04	45.24	79.91	51.81	5.99	39.20
	Corn straw	92.42	95.20	0.36	3.05	39.67	79.86	47.75	4.80	44.55
	Barley straw	92.80	95.84	0.57	5.40	29.46	57.40	33.09	4.16	53.20
Mean		92.54	93.35	0.75 ^b^	6.19 ^b^	39.89 ^a^	73.22 ^a^	46.18 ^a^	6.65	39.06 ^b^
SEM ^1^	Forage vs. Crop residues	0.34	0.59	0.15	1.42	1.95	3.79	2.15	0.59	0.14
*p* value	Forage vs. Crop residues	0.94	0.27	<0.01	<0.01	<0.01	<0.01	<0.01	0.27	<0.01

^1^ SEM: standard error of the mean. Means within the same list with different letters (a, b) differ (*p* < 0.05).

**Table 2:** *In Situ* organic matter degradation characteristics of roughages.

Item	Roughages	a, %	b, %	c, %h	FOM*_in situ_*, % ^1^
Forages	Langsdorff small reed	20.84	54.16	0.017	30.55
	Alfalfa	24.48	58.03	0.045	41.13
	Mix forage	21.22	52.65	0.029	64.36
	Lolium perenne	21.33	50.90	0.022	47.33
	*Poa annua L.*	20.06	50.77	0.020	50.9
	*Alpine kobresia*	19.01	52.44	0.010	41.02
Mean		21.16 ^a^	53.16 ^a^	0.024 ^a^	45.88 ^a^
Crop residues	Oat straw	20.61	48.36	0.018	38.85
	Hulless barley straw	19.05	49.43	0.016	27.51
	Rapeseed straw	19.37	48.40	0.019	29.79
	Avena nuda straw	19.93	48.43	0.017	26.14
	Corn straw	19.25	50.90	0.012	33.72
	Barley straw	19.90	50.48	0.010	49.26
Mean		19.69 ^b^	49.33 ^b^	0.015 ^b^	34.21 ^b^
SEM ^2^	Forages vs. Crop residues	0.48	0.70	0.003	3.37
*p* value	Forages vs. Crop residues	<0.01	<0.01	0.01	<0.01

^1^ FOM*_in situ_*: fermentable organic matter determined by *in situ* nylon bag technique. ^2^ SEM: standard error of the mean. Means within the same list with different letters (a, b) differ (*p* < 0.05).

**Table 3:** Prediction equations between FOM*_in situ_* and chemical compositions of roughages.

Item	Regression Equation ^1^	R^2^	*p*-Value	RMSE ^2^
1	FOM*_in situ_* = 88.85 − 1.40 CF	0.84	<0.01	4.76
2	FOM*_in situ_* = 89.52 − 1.22 ADF	0.87	<0.01	4.25
3	FOM*_in situ_* = 78.62 + 6.67 EE − 1.27 CF	0.92	<0.01	3.65
4	FOM*_in situ_* = 89.79 + 5.44 EE − 0.44 CP − 1.26 ADF	0.92	<0.01	3.90
5	FOM*_in situ_* = 84.80 + 8.56 EE − 0.39 CP − 0.64 CF	0.93	<0.01	3.48

^1^ FOM*_in situ_*: fermentable organic matter determined by *in situ* nylon bag technique. ^2^ RMSE: root mean squared error.

**Table 4:** Comparison between FOM_NDC_ and FOM*_in situ_* of roughages (DM basis, expressed as %).

Item ^1^	Roughages	FOM_NDC_ ^1^	CV ^2^	FOM*_in situ_* ^3^	CV	SEM ^4^	*p*-Value
Forages	Langsdorff small reed	28.76	1.83	30.55	12.99	1.63	0.48
	Alfalfa	47.77 ^a^	1.64	41.13 ^b^	6.56	1.15	0.02
	Mixed forage	71.43	1.73	64.36	9.09	2.44	0.11
	Lolium perenne	45.70	4.30	47.33	4.91	1.24	0.41
	*Poa annua*	43.96	2.70	50.90	12.76	2.69	0.14
	*Alpine kobresia*	41.99	1.69	41.02	9.29	1.58	0.69
Crop residues	Oat straw	38.49	3.80	38.85	13.44	2.21	0.91
	Hulless barley straw	21.60	3.61	27.51	17.99	2.04	0.11
	Rapeseed straw	31.96	4.36	29.79	5.48	0.88	0.15
	Avena nuda straw	23.86	4.24	26.14	5.68	0.73	0.09
	Corn straw	32.44	5.41	33.72	9.38	1.48	0.57
	Barley straw	46.83	3.23	49.26	7.34	1.60	0.34

^1^ FOM_NDC_: fermentable organic matter determined by *in vitro* neutral detergent–cellulase plus amylase method. ^2^ CV: coefficient of variation. ^3^ FOM*_in situ_*: fermentable organic matter determined by *in situ* nylon bag technique. ^4^ SEM: root mean squared error. Means within the same row with different letters (a, b) differ (*p* < 0.05).

**Table 5:** Prediction equations between FOM_NDC_ and chemical compositions of roughages.

Item	Regression Equation ^1^	R^2^	*p*-Value	RMSE ^2^
1	FOM_NDC_ = 96.31 − 1.60 CF	0.79	<0.01	6.51
2	FOM_NDC_ = 81.99 + 9.32 EE − 0.76 NDF	0.83	< 0.01	6.10
3	FOM_NDC_ = 96.83 − 1.41 ADF	0.84	<0.01	5.59
4	FOM_NDC_ = 88.74 + 10.82 EE − 0.34 CP − 0.84 NDF	0.84	<0.01	6.33
5	FOM_NDC_ = 85.47 + 5.79 EE − 1.27 ADF	0.88	<0.01	5.18
6	FOM_NDC_ = 86.20 + 5.96 EE − 0.04 CP − 1.28 ADF	0.88	<0.01	5.49
7	FOM_NDC_ = 80.73 + 9.03 EE + 0.02 CP − 1.41 CF	0.89	<0.01	5.31
8	FOM_NDC_ = 80.98 + 9.10 EE − 1.41 CF	0.89	<0.01	5.00

^1^ FOM_NDC_: rumen fermentable organic matter digestibility determined by *in vitro* neutral detergent–cellulase plus amylase method. ^2^ RMSE = root mean squared error.

## Data Availability

All the data are already provided in the main manuscript.
